# Evaluation of the *in vitro* acaricidal effect of five organic compounds on the cattle fever tick *Rhipicephalus* (*Boophilus*) *microplus* (Acari: Ixodidae)

**DOI:** 10.1007/s10493-023-00780-9

**Published:** 2023-04-13

**Authors:** Adela S. Oliva Chávez, Stephanie Guzman Valencia, Geoffrey E. Lynn, Charluz Arocho Rosario, Donald B. Thomas, Tammi L. Johnson

**Affiliations:** 1 Department of Entomology, Texas A&M University, 77845 College Station, TX USA; 2 Texas A&M AgriLife Research, 78801 Uvalde, TX USA; 3 Cattle Fever Tick Research Laboratory, USDA-ARS, 78541 Edinburg, TX USA; 4 Department of Rangeland, Wildlife and Fisheries Management, Texas A&M University, 78801 Uvalde, TX USA

**Keywords:** Repellent, Tick management, Acaricide, Ticks, Plant-derived compounds

## Abstract

The cattle fever tick, *Rhipicephalus* (*Boophilus*) *microplus*, is the most economically important tick worldwide. Infestations with this tick can lead to direct damage and cattle mortality due to the transmission of potentially deadly pathogens. Management of this tick species has been focused on the use of synthetical acaricides; however, the emergence of acaricide resistance to single or multiple active ingredients has resulted in a need for novel acaricide compounds. Among potential avenues for the discovery of novel acaricides are plant-derived compounds. The efficacy of five organic compounds (nootkatone, Stop the Bites®, BioUD®, lavender oil, and cedarwood oil) was evaluated using larval immersion tests (LITs), repellency assays, and adult immersion tests (AITs). The results from the LITs indicate that three of the organic compounds (NootkaShield™, Stop the Bites, BioUD) led to significant mortalities at low concentrations (0.2, 0.02, and 0.08%, respectively). By comparison, lavender and cedar oil led to around 90% mortality at 10 and 1% concentrations, respectively. Similarly, NootkaShield, Stop the Bites, and BioUD had strong repellent properties with over 90% repellency at the two highest concentrations tested. Using the FAO 2004 guidelines, we evaluated the effectiveness of these organic compounds at reducing the fecundity of *R.* (*B.*) *microplus* and show that Nootkatone, Stop the Bites, and BioUD may significantly decrease tick populations (Drummond’s index > 90% at concentrations of 5%), highlighting their potential as alternatives to synthetic acaricides for the control of cattle fever ticks.

## Introduction

The US cattle industry is constantly threatened with the introduction and spread of potentially fatal pathogens that can cause diseases, such as bovine babesiosis. Coinfections of *Babesia bigemina/Babesia bovis* and other tick-borne pathogens such as *Anaplasma marginale* and *Borrelia theileri* have been reported in cattle crossing the border between Texas (USA) and Mexico (Scoles et al. 2021) and are transmitted by *Rhipicephalus* (*Boophilus*) *microplus*, commonly known as the cattle fever tick. The potential threat posed by this tick species to the multi-million USD cattle trade between USA and Mexico, and the efforts to manage or eradicate *R.* (*B.*) *microplus* were the topic of discussion by several experts from USA and Mexico (Esteve-Gasent et al. 2020). The control of the cattle fever tick has relied on the use of synthetic chemical acaricides (Barré et al. 2008; Brito et al. 2011); however, due to the extensive use, improper dosing, and the inadequate rotation of acaricides, ticks have developed tolerance, cross-resistance, and multi-acaricide resistance to common and conventional acaricides used for tick control (Esteve-Gasent et al. 2020). Consequently, potential safe alternatives have been developed to control acaricide resistant cattle fever tick populations (Camargo et al. 2014; de Mendonça et al. 2019; Khan et al. 2019; Shapiro-Ilan and Goolsby 2021).

Plant-derived compounds have shown promising acaricidal activity against different tick species (Quadros et al. 2020). For example, lavender (*Lavandula* spp.) essential oil caused mortalities between 73 and 100% in *Rhipicephalus* (*Boophilus*) *annulatus* with in vitro experiments (Pirali-Kheirabadi and Teixeira da Silva 2010) but showed low repellent activity against *Ixodes ricinus* (Kröber et al. 2013). Likewise, cedarwood oil has demonstrated acaricidal activity against *Ixodes scapularis* nymphs (Eller et al. 2014). Nootkatone is a natural organic compound derived from various sources such as *Chamaecyparis nootkatensis* and *Citrus grandis* (Flor-Weiler et al. 2011; Quadros et al. 2020). NootkaShield™ (Evolva) has recently been approved by the Environmental Protection Agency (EPA) for the prevention of tick bites (CDC 2020). Nootkatone leads to efficient control of *I. scapularis* in both short- and long-term conditions during field trials, depending on the formulation (Behle et al. 2011; Bharadwaj et al. 2012). Further, this compound has shown toxicity against other tick species such as *Rhipicephalius sanguineus, Dermacentor variabilis*, and *Amblyomma americanum* (Dolan et al. 2009; Flor-Weiler et al. 2011) and presents repellent activity against *I. scapularis* and *A. americanum* (Jordan et al. 2012; Schulze et al. 2011).

Other commercial anti-arthropod plant-derived products are available in the market. BioUD® (7.75% 2-undecanone, Bite Blocker, HOMS) is an organic compound derived from *Lycopersicon hirsutum*. This compound exhibited a similar repellency activity to *D. variabilis* when compared with conventional repellents, such as DEET (Witting-Bissinger et al. 2008), and it was also repellent for *A. americanum* (Bissinger et al. 2011) and *I.* *scapularis* (Bissinger et al. 2009). Lastly, Stop the Bites® by Arkion Life Sciences is promoted as an eco-friendly outdoor product that kills and controls ticks in the lawn (Arkion). The main ingredient, lemongrass oil, induces partial control (39%) of *R.* (*B.*) *microplus* at 2% (Agnolin et al. 2014), and at high concentrations (40–80 mg/ml), high mortality was reported in *Haemaphysalis longicornis* (Agwunobi et al. 2020). Castor oil, another component of Stop the Bites, reduces egg production of *R. sanguineus* (Arnosti et al. 2011), and leads to mortalities between 55 and 95% in *R.* (*B.*) *microplus* (Ghosh et al. 2013). The objective of this study was to assess the in vitro performance of five plant-derived products: NootkaShield, lavender oil, Stop the Bites, BioUD, and cedarwood oil against *R.* (*B.*) *microplus*. Although these compounds have previously been tested in other tick species, no information is available about their efficacy on cattle fever ticks. Herein, we demonstrate that the commercially available products (NootkaShield, Stop the Bites, and BioUD) are potential alternatives for the control of *R.* (*B.*) *microplus* as repellents and acaricides.

## Materials and methods

### Ticks

All the experiments were performed using *R.* (*B.*) *microplus* Deutsch strain, from colonies maintained at the Cattle Fever Tick Research Laboratory (CFTRL), Edinburg, TX, USA (Tidwell et al. 2021). The Deutsch strain is an acaricide-susceptible reference strain that has been used as comparison to acaricide-resistant ticks (Klafke et al. 2019). Larval ticks from the same oviposition date were used with all the different compounds and controls to reduce variability in LITs. Engorged females were collected on the detachment day, rinsed, and visually examined to ensure that ticks were not damaged. Ticks were subjected to treatment on the following day.

### Compounds

Nootkatone (NootkaShield NKS226) [20% working concentration] (Evolva, Reinach, Switzerland), Stop the Bites [20% working concentration] (Arkion Life Sciences, New Castle, DE, USA), BioUD [7.75% working concentration] (HOMS, Pittsboro, NC, USA), lavender oil [100% working concentration] (Natures-Star, New York, NY, USA), and cedarwood oil [100% working concentration] (Texas Cedar Oil, Uvalde, TX, USA).

### Larval immersion tests (LITs)

Larval Immersion Tests (LITs) were performed as previously described in Shaw (1966) with modifications described in Klafke et al. (2012). Briefly, all the compounds were diluted with RO water plus 0.05% Triton X-100 (Thermo Fisher Scientific, Waltham, MA, USA), which was previously tested to confirm that a homogenous solution was obtained at all tested concentrations. NootkaShield (NKS226) was diluted to 4, 2, 1, 0.5, 0.2, and 0.02%; Stop the Bites was diluted to 2, 0.2, 0.02, 0.004, 0.0024, 0.002, 0.0014, 0.00086, and 0.0005%; BioUD was diluted to 0.2, 0.08, 0.032, 0.02, 0.0128, 0.005, and 0.002%; the lavender oil was diluted to 10, 1, 0.1, and 0.01%; and the cedarwood oil was diluted to 10, 1, 0.1, and 0.01%. These concentrations were set based on initial tests using 10-fold dilutions starting from the concentration recommended by the manufacturer. In the case of lavender oil and cedarwood oil, a 10-fold dilution starting at 10% was used because no recommendations were available. This method has been suggested by the FAO (2004) guidelines and validated previously at the CFTRL (Jonsson et al. 2007). A negative control was included for each compound consisting of RO water + 0.05% Triton X-100 only. Approximately, 100 larvae were submerged in 1 ml of the dilution for 10 min under constant agitation. Treatments were done simultaneously in triplicates. After treatment, ticks were pipetted out of the liquid onto filter paper to remove the excess of liquid and placed inside 8 × 9 cm filter paper Whatman No. 1 packets (Whatman, Madstone, UK), using metal clips. The packets were maintained for 24 h at 28 °C, 70–80% RH under at 12:12 L:D photoperiod. After 24 h, the packets were opened, and the live/dead ticks were counted. Dead ticks were determined by the lack of movement even when stimulated with a brush as well as signs of desiccation. Statistical differences between treatments and concentrations were evaluated using a one-way ANOVA followed by Tukey’s test for multiple comparisons of the mean of each treatment with GraphPad Prism v.9.4.0 (GraphPad Software, San Diego, CA, USA).

### Repellency bioassay

**Fig. 1:**
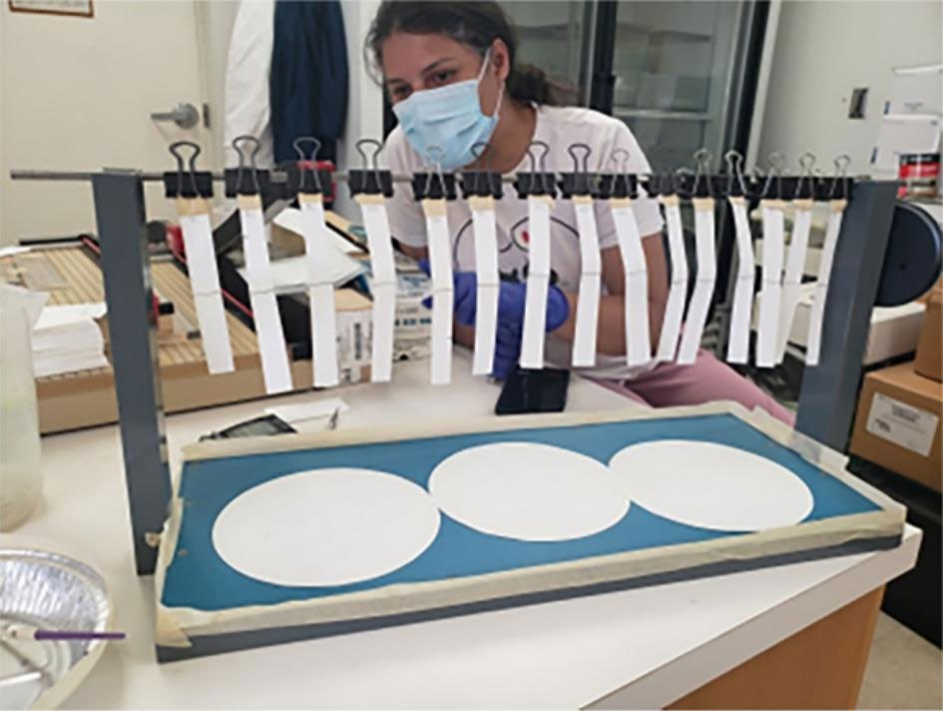
Experimental procedures performed during the repellent assays. Repellency assays (trapeze test) consisted of paper strips suspended from a horizontal bar, each treated with different concentrations of the organic compounds applied at the midline. Larval ticks were placed at the bottom of each strip and allowed to ascend naturally. The numbers surpassing or not passing the midline were assessed for each strip

The repellency of the organic compounds was tested using trapeze tests (Fig. 1). Filter paper was cut in strips of 2 × 15 cm and a line was drawn across the middle using a graphite pencil. Organic compounds were diluted in RO water with 0.05% Triton-X 100 as follows: NootkaShield (NKS226) = 3, 2, and 1%; Stop the Bites = 2.5, 1.25, and 0.625%; BioUD = 7.75, 3.875, and 1.9375%; lavender oil = 10, 1, and 0.1%; and cedarwood oil = 10, 1, and 0.1%. These concentrations were used following manufacturer’s recommendations for the commercial compounds. Fifty µl of each dilution was applied to the middle of the strip (at the pencil line). Controls consisted of strips treated with 0.05% Triton-X 100 in RO water, or not treated at all (blank). The strips were allowed to dry for 20 to 30 min inside a fume hood and were suspended in the trapeze as shown in Fig. 1. Around 10 larvae were transferred to the bottom of each strip with a paint brush. The ticks crossing the middle of the strip at 1, 5, 10, 20, and 30 min were counted. The level of repellency was calculated using the formula:

$$\% {\text{ }} Repellency = \,[\frac{{Total{\text{ }}no.\,ticks{\text{ }}applied{\text{ }}to{\text{ }}the{\text{ }}strip\, - \,No.{\text{ }}ticks{\text{ }}crossed{\text{ }}middle{\text{ }}point\, }}{{Total{\text{ }}no.\,ticks{\text{ }}applied{\text{ }}to{\text{ }}the{\text{ }}strip}}] * 100.$$

Each compound was tested in triplicate simultaneously. The experiment was repeated twice with newly prepared dilutions of the compounds and batches of larvae. Statistical differences between treatments and concentrations were evaluated using a one-way ANOVA followed by Tukey’s test for multiple comparisons of the mean of each treatment with GraphPad Prism v.9.4.0.

### Adult immersion test (AIT)

Adult immersion tests (AITs) were performed as described in Peniche-Cardeña et al. (2022), with slight modifications. Organic compounds were diluted in 0.1% Triton X-100 with RO water as follow: NootkaShield (NKS226) = 5, 2.5, 1.25, and 0.625%; Stop the Bites = 5, 2.5, 1.25, and 0.625%; BioUD = 5, 2.5, 1.25, and 0.625%; lavender oil = 20, 10, 5, and 2.5%; and cedarwood oil = 20, 10, 5, and 2.5%. Control consisted of 0.1% Triton X-100 with RO only. Females that were not damaged during collection or cleaning were weighed in groups of 10 (three groups/compounds = 30 ticks) and submerged in 10 ml of diluted organic compounds or control (each compound had its own control) for 30 min at constant agitation. Treated females were dried on paper towels and placed on Petri dishes (100 × 15 mm) containing filter paper (Whatman No. 1). The Petri dishes were maintained at 28 °C, 70–80% RH under at 12:12 L:D photoperiod for 14 days until ticks finished oviposition, at which point the females with eggs were counted. Those females without eggs were considered dead and counted to calculate mortality rates. The eggs were separated from the females, weighed, and placed inside glass vials with cotton plugs. Egg masses were maintained at 28 °C, 70–80% RH under at 12:12 L:D photoperiod until hatching was finished and the percentage of hatching based on proportion of larvae to unhatched eggs was visually estimated with the aid of a dissecting stereoscope (Davey et al. 2013). The accuracy (precision) of the visual estimations has been confirmed previously through full count comparisons in our laboratory (data not shown). To reduce potential variations a single individual with prior training and experience performed all the hatching estimations for all the assays. This approach has been evaluated in *R.* (*B.*) *microplus* using strains from the USA and Australia (Jonsson et al. 2007). The effects of the compounds on mortality and hatching were evaluated by one-way ANOVA, followed by a Tukey’s test for multiple comparisons of the mean of each treatment with GraphPad Prism v.9.4.0. The mortality was calculated using the formula:

$$\% {\text{ }}Survival{\text{ }} = {\text{ }}[\frac{{Total{\text{ }}no.{\text{ }}females{\text{ }}plated - No.{\text{ }}females{\text{ }}without{\text{ }}oviposition}}{{Total{\text{ }}no.{\text{ }}females{\text{ }}plated}}]*100.$$

The percent hatch was estimated as described above. The overall effect of the compounds on tick reproduction was calculated using Drummond’s equation (Drummond et al. 1973):

$$\begin{gathered} Estimated\,reproduction\,(ER)\, = \,[\frac{{Weight\,eggs}}{{Weight\,females}}]*estimated\,\% \,hatch\,*\,20,000,\,{\text{and}} \\ \,\% \,Control = \left[ {\frac{{\sum {ER\,untreated - \sum {ER\,treated} } }}{{\sum {ER\,untreated} }}} \right]*100. \\ \end{gathered}$$

## Results

### Anti-larval efficacy

**Fig. 2:**
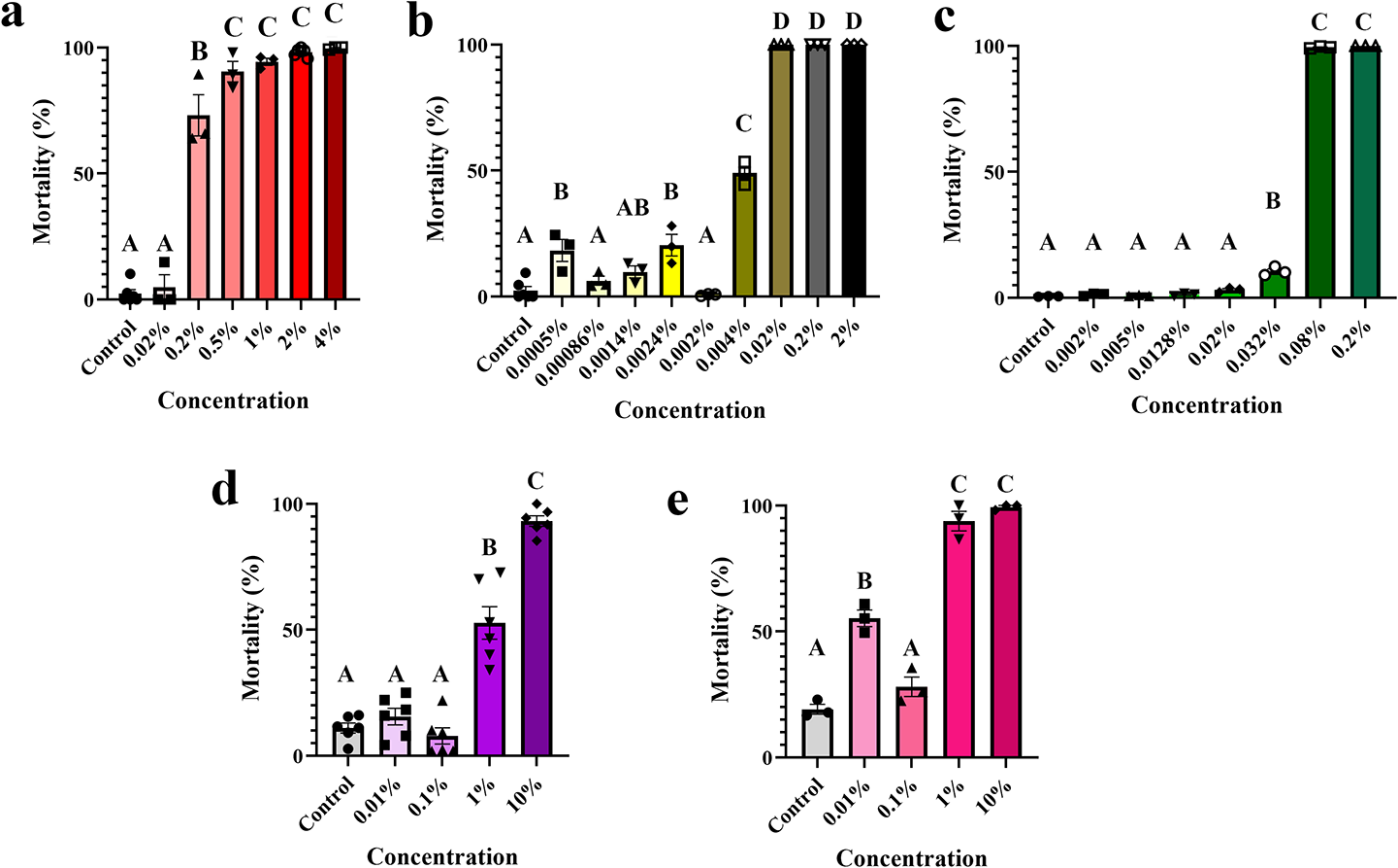
Mean (+ SEM) acaricidal effect (% mortality) of organic compounds on *Rhipicephalus* (*Boophilus*) *microplus* larvae. Mortality of larvae after LITs evaluation of acaricidal effect of (a) NootkaShield, (b) Stop the Bites, (c) BioUD, (d) lavender oil, and (e) cedarwood oil. Means within a panel capped with different letters are significantly different (p ≤ 0.05). Graphs show the result of one representative experiment from two to three different experiments comprised of three biological replicates (ca. 100 larvae per replicate)

All compounds tested had acaricidal effects on larvae at low concentrations. Stop the Bites and BioUD presented the highest acaricidal activity.  Concentrations as low as 0.02 and 0.08% led to around 90% mortality, respectively, and statistically significant mortalities starting at 0.004 and 0.032%, respectively (p<0.0001). By comparison, larval treatment with NootkaShield resulted in significant mortalities starting at 0.2% (p<0.0001). Cedar and lavender, on the other hand, showed weaker acaricidal impacts (Fig. 2). These in vitro test results indicate that Stop the Bites, BioUD, and NootkaShield could potentially be used for the control of *R.* (*B.*) *microplus* larvae. These results suggest that low concentrations of NootkaShield, Stop the Bites, and BioUD are effective at killing larval *R.* (*B.*) *microplus *(Fig. 2).

### Repellency effect

**Fig. 3:**
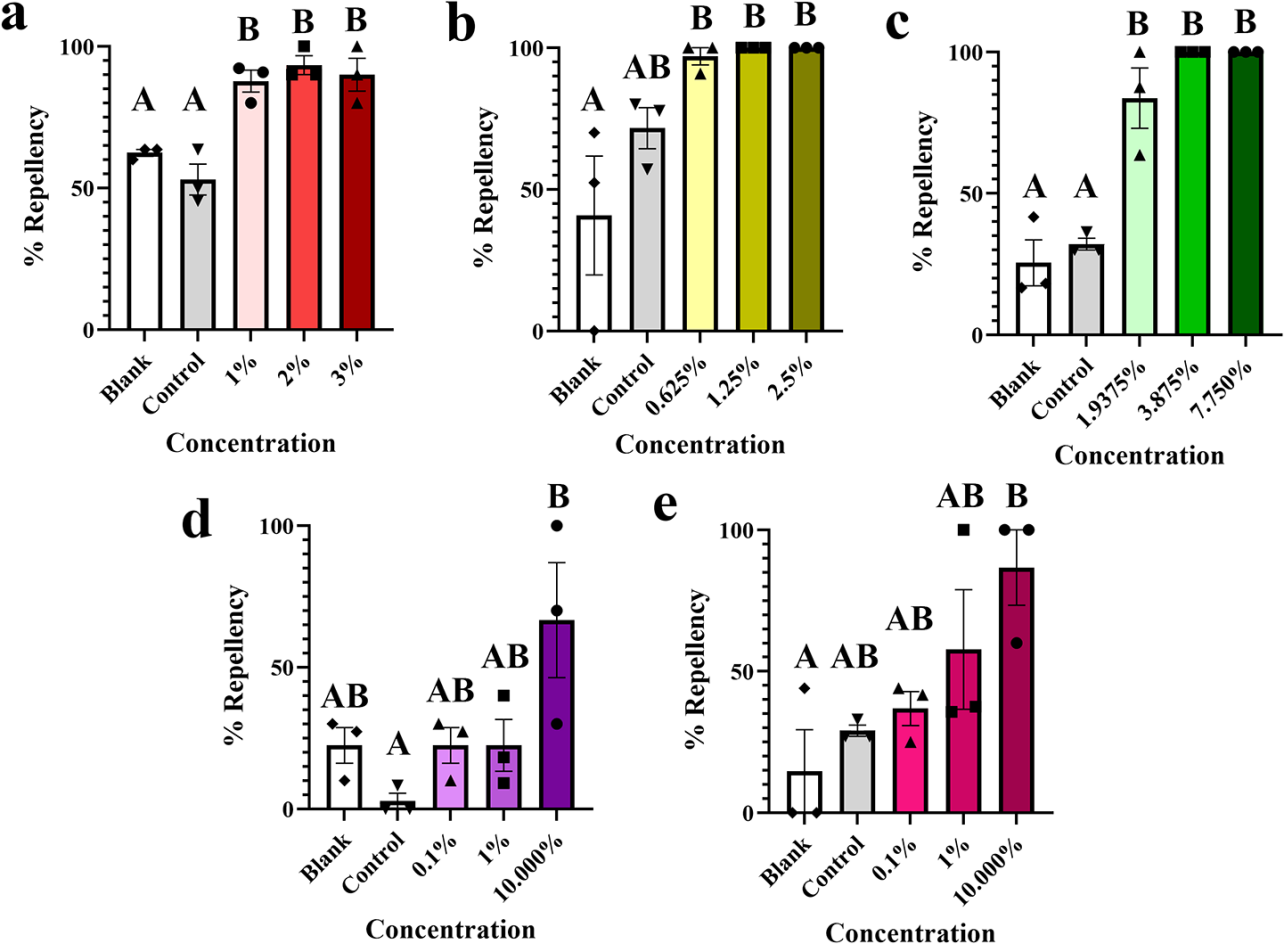
Mean (+ SEM) repellent effect (% repellency) of organic compounds on *Rhipicephalus* (*Boophilus*) *microplus* larvae. Repellency trapeze assays of (a) NootkaShield, (b) Stop the Bites, (c) BioUD, (d) lavender oil, and (e) cedarwood oil. Means within a panel capped with different letters are significantly different (p ≤ 0.05). Graphs show the result of one representative experiment from two different experiments

We assessed the repellency of five organic compounds to *R.* (*B.*) *microplus* larvae at different concentrations using trapeze assays (Fig. 1). All three concentrations of NootkaShield (NKS226) [3, 2, and 1%] were more repellent than the blank (p ≤ 0.012; Fig. 3a). Similarly, all concentrations of Stop the Bites [2.5, 1.25, and 0.625%] and BioUD [7.75, 3.875, and 1.9375%] had repellent properties (p ≤ 0.018 and p ≤ 0.0009, respectively; Fig. 3b-c). In most of the cases, these compounds had > 90% repellency (Fig. 3a-c). Lavender and cedarwood oil, on the other hand, were only repellent to *R.* (*B.*) *microplus* larvae in the highest concentration [10%] (p < 0.05; Fig. 3d-e). Nevertheless, the average repellency of cedarwood oil at 10% was only 86.7% and lavender was 66.7%, confirming that these compounds were in fact weaker than the commercially available repellents. The control (0.05% Triton X-100 in RO water) was not significantly repellent in any of the assays (Fig. 3), confirming that the effect observed was due to the organic compounds. According to these results, all commercially available compounds can efficiently repel *R.* (*B.*) *microplus* larvae.

### Adult immersion test

**Fig. 4:**
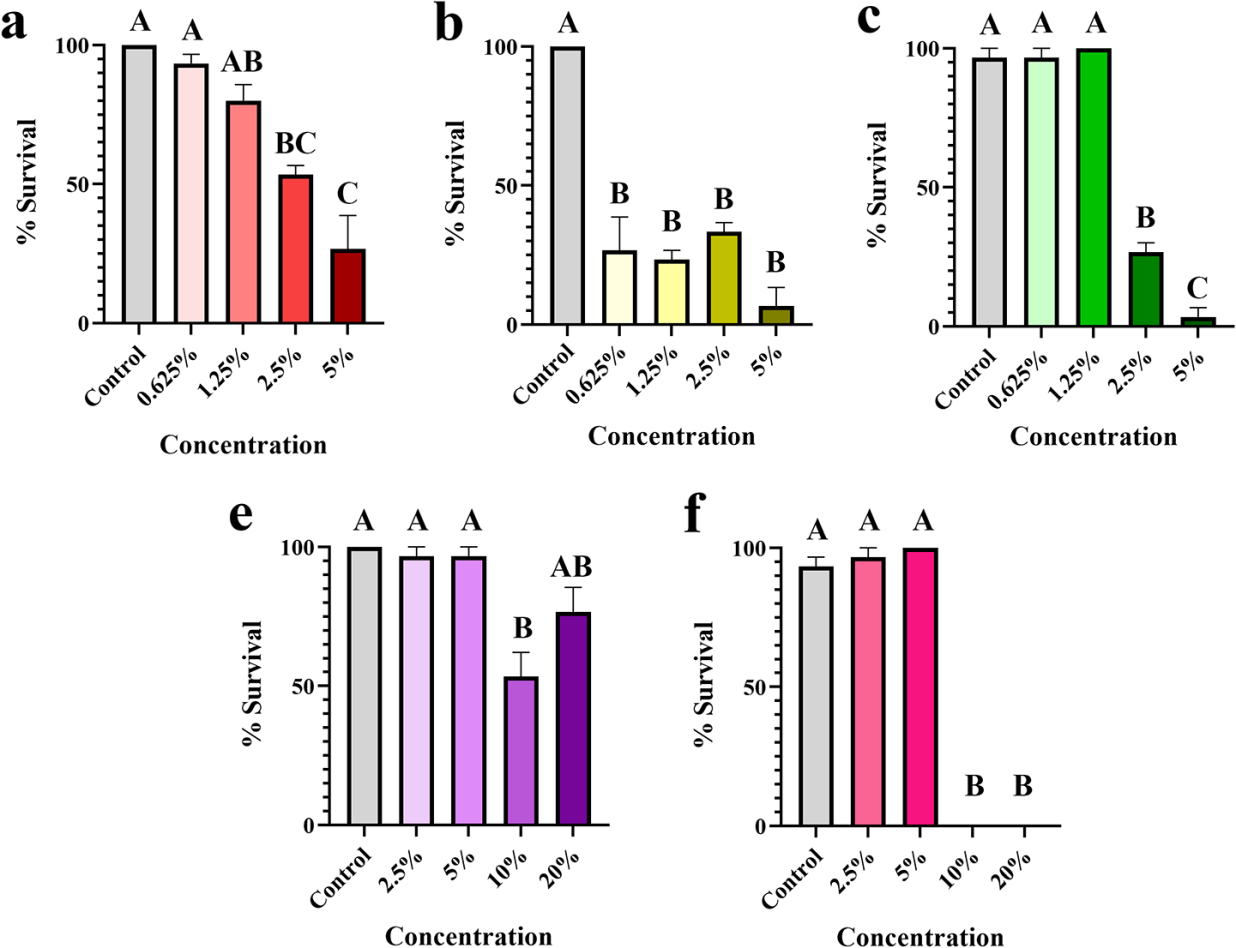
Mean (+ SEM) survivorship (%) of *Rhipicephalus* (*Boophilus*) *microplus* females after treatment with organic compounds. *Rhipicephalus* (*B.*) *microplus* were treated for 30 min with different concentrations of (a) NootkaShield, (b) Stop the Bites, (c) BioUD, (d) lavender oil, or (e) cedarwood oil. Females were allowed to lay eggs and surviving females were determined by successful oviposition. Means within a panel capped with different letters are significantly different (p ≤ 0.05). Graphs show the results from one experiment comprised of three biological replicates (three groups of 10 females; n = 30 females)

**Fig. 5:**
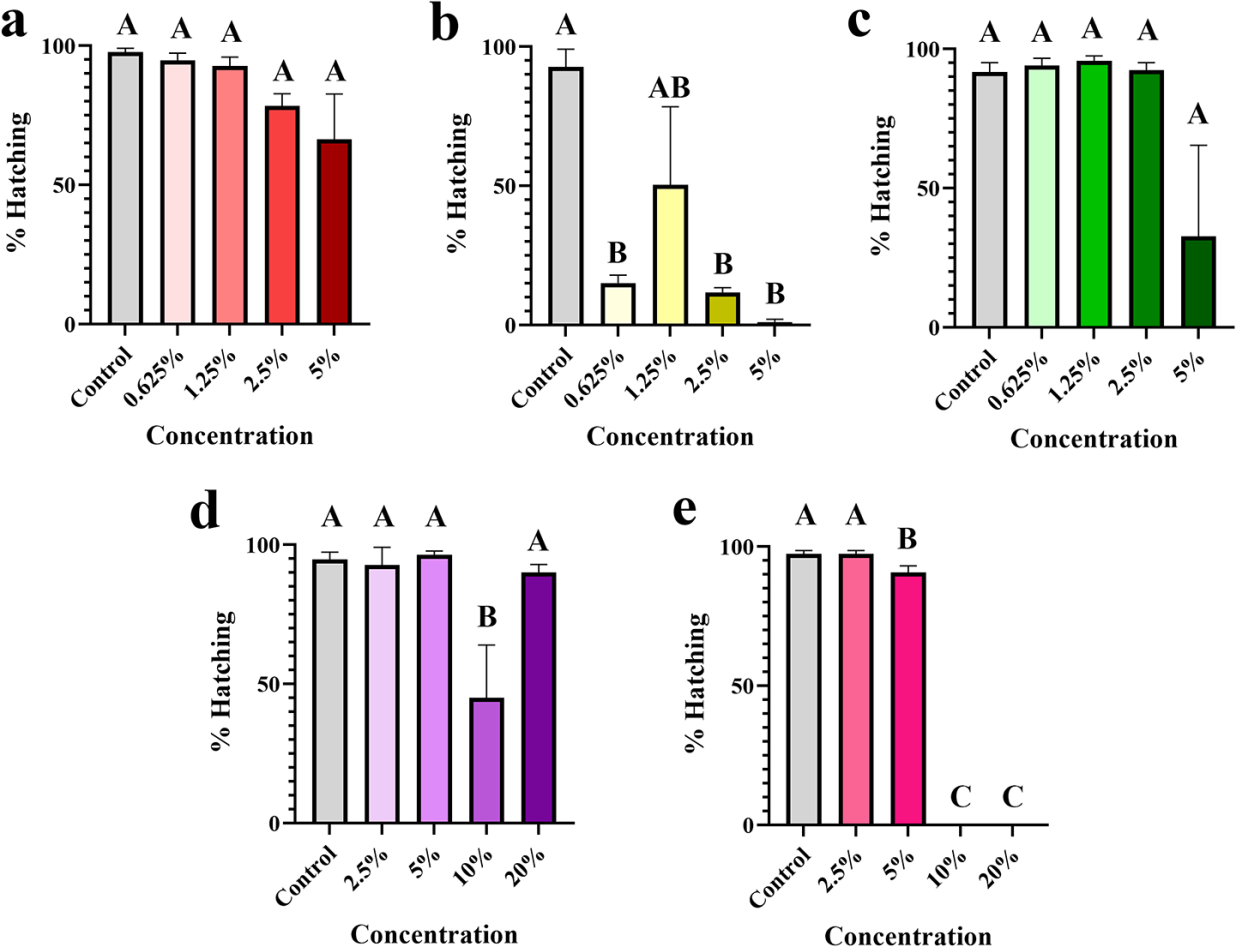
Mean (+ SEM) egg hatching (%) of *Rhipicephalus* (*Boophilus*) *microplus* treated with organic compounds. The effect of (a) NootkaShield, (b) Stop the Bites, (c) BioUD, (d) lavender oil, and (e) cedarwood oil on egg eclosion was evaluated 28 days after treatment of female *R.* (*B.*) *microplus.* Eggs were collected after females completed oviposition (14 days after treatment) and placed into glass vials. Eggs were maintained at high relative humidity for 14 days and % hatching was estimated based on the proportion of larvae to unhatched eggs. Means within a panel capped with different letters are significantly different (p ≤ 0.05). Graphs show the results from one experiment comprised of three biological replicates (three groups of 10 females; n = 30 females)

We tested the effect of five organic compounds on the survival, egg hatching, and the reproductive index of *R.* (*B.*) *microplus* female ticks using AIT at different concentrations. Interestingly, all the concentrations tested for Stop the Bites had a strong acaricidal effect on fully fed *R.* (*B.*) *microplus* females (p ≤ 0.0001; Fig. 4b). NootkaShield and BioUD, on the other hand, resulted in significant mortalities at 2.5 and 5% (p ≤ 0.0028 and p < 0.0001, respectively; Fig. 4a and 4c). Lavender and cedarwood oil were acaricidal only at high concentrations (10–20%), with 50–100% mortalities, respectively (p ≤ 0.0031; Fig. 4d-e). Similarly, all concentrations of Stop the Bites, except 1.25%, led to a reduction in egg hatching (p ≤ 0.0115; Fig. 5b), whereas neither NootkaShield nor BioUD had a significant effect (Fig. 5a-b). Stop the Bites at 0.625, 2.5, and 5% resulted in only 15, 11.6, and 1% hatching, respectively (Fig. 5b). Lavender oil had a variable effect on hatching with 10% lavender oil causing a reduction in hatching (45% hatching; p = 0.021). None of the other concentrations tested resulted in significant reductions in egg hatching (Fig. 4d). Cedarwood oil, in contrast, reduced egg hatching at 5–20% concentrations (p ≤ 0.029; Fig. 5e).

These results are reflected in the reduction of the reproductive success of *R.* (*B.*) *microplus* obtained for each compound. Stop the Bites caused significant reductions (96–99.9%) in the reproductive success of *R.* (*B.*) *microplus* females at all concentrations. Conversely, although 2.5% NootkaShield and BioUD significantly reduced the survival of treated *R.* (*B.*) *microplus* females (Fig. 3a), the lack of effect on egg hatching led to poor control, according to the Drummonds index (control 73% and 79.4, respectively; Table 1). Likewise, lavender oil and cedarwood oil only decreased the reproductive potential of *R.* (*B.*) *microplus* at high concentrations (> 5%; Table 1). Our results suggest that in vitro treatment with low concentrations of Stop the Bites leads to high mortality, decreased egg hatching, and significant reduction in reproductive success of *R.* (*B.*) *microplus.*

**Table 1:** Control (%) of *Rhipicephalus* (*Boophilus*) *microplus* reproductive success of NootkaShield, Stop the Bites, BioUD, lavender oil, and cedarwood oil

Compound	Drummond’s index (% control)				
	Control*	0.63%	1.25%	2.5%	5%
Nootkatone	0	14.5	32.26	73	95
Stop the Bites	0	98.5	96	98.97	99.9
BioUD	0	6.2	-2.9	79.4	95.97
Lavender oil	0	-1.14	-0.009	76	44.76
Cedarwood oil	0	-7.23	-11.57	100	100

*Controls represent 0% concentration of the compounds (0.1% Triton X-100 with RO water). The percentages next to the control represent the concentrations for Nootkatone, Stop the Bites, and BioUD. 2.5% for lavender and cedar oil. 5% for lavender and cedar oil. 10% for lavender and cedar oil. 20% for lavender and cedar oil.

## Discussion

Plant-derived acaricides or biopesticides are particularly compelling as alternatives to conventional acaracides for tick management. Nevertheless, difficulties in regulatory approval by governmental organizations can delay the commercialization of several compounds of interest (Quadros et al. 2020). Thus, acaricides based on plant-derived compounds that have received approval for the management of other tick species are of particular interest for cattle fever control programs such as the Cattle Fever Tick Eradication Program (CFTEP) in Texas (Esteve-Gasent et al. 2020; Graham and Hourrigan 1977).

We tested the acaricidal effect of three commercially available repellents and acaricides [NootkaShield (CDC 2020), Stop the Bites (Arkion), and BioUD (Witting-Bissinger et al. 2008)] and two essential oils (lavender and cedarwood oil). Stop the Bites, and to a lesser extent NootkaShield and BioUD, had a significant impact on the survival and reproduction of *R.* (*B.*) *microplus* larvae and females (Fig. 2 and Table 1). As mentioned above, Stop the Bites is a mixture of several essential oils that have previously shown to be effective against other tick species (Arnosti et al. 2011; Agnolin et al. 2014; Agwunobi et al. 2020). Among the essential oils present in Stop the Bites, castor oil (Arkion), which is produced from *Ricinus communis*, has been previously shown to have acaricidal properties against acaricide resistance *R.* (*B.*) *microplus* populations leading to 48–56.7% mortalities (Ghosh et al. 2013). Whether Stop the Bites can be effectively used for the control of acaricide-resistant ticks remains to be determined but our results suggest that this compound may be a viable alternative to synthetical acaricides. Likewise, whether the results of our in vitro test translate in effective tick control in animal applications needs to be addressed.

Stop the Bites is patented for applications in turf and outdoors. This product contains a combination of 19% essential oils (castor oil, lemon grass oil, geraniol, cedarwood oil, and corn oil), 1% sodium lauryl sulfate, and 80% inert ingredients (Arkion). Therefore, whether this product could be used for on-host applications without stressing or repelling animals was unknown until recently. A recent study by Goolsby et al. (2022) evaluated whether ground or direct spray applications of Stop the Bites from remotely operated sprayers would deter deer from approaching a corn feeder. According to their results, the application of the commercial doses (20%) of Stop the Bites did not significantly repel deer from consuming corn from treated feeders when compared to the non-treatment controls. Our data indicates that concentrations as low as 2.5% (10× less than the concentration used by Goolsby et al. 2022) can effectively control *R.* (*B.*) *microplus* ticks (Fig. 3; Table 1), suggesting that the dosages of 2.5–5% could potentially be used for the control of the cattle fever tick in wild deer populations. The incursion of deer and nilgai infested with cattle fever ticks into protected areas within southern coastal areas in Texas has threatened the management efforts by the CFTEP. Current control strategies have included the use of ivermectin treated corn within feeders (Thomas and Duhaime 2022). However, a withdrawal period during hunting season may affect control efforts. Thus, plant-derived organic compounds that can be used for the management of cattle fever ticks during these periods will have the potential to augment current efforts (Goolsby et al. 2022). Future studies will evaluate the efficacy of this and other organic compounds for the control of ticks in vivo by applying the compounds directly on infested animals.

The two essential oils tested (lavender and cedarwood oil) had only modest effects, relative to the other compounds we evaluated (Table 1). These oils affected adult tick survival only at concentrations of 10% or higher (Fig. 4d-e). Our results contrast with those of Pazinato et al. (2016) who reported 0% hatching success of eggs masses from *R.* (*B.*) *microplus* females treated with 1, 5, and 10% cedarwood oil. However, lack of acaricidal effects of cedarwood oil have previously been reported for *A. americanum* nymphs (Flor-Weiler et al. 2022). The differences in effect may be due to the plant source used to produce the essential oil. As reported by Flor-Weiler et al.(2002), the cedarwood oil tested herein is produced from juniper trees, although a different species of juniper (Mexican juniper, *Juniperus ashei*; Texas Cedar Oil, http://www.texascedaroil.com, accessed 8/28/2022), whereas Pazinato et al. (2016) tested oil from Atlas cedar (*Cedrus atlantica*). Lavender oil had not been tested as an acaricide against ticks; however, its repellency against *I. ricinus* had been assessed previously, showing repellent properties only at 30% (Jaenson et al. 2006). In our study *R.* (*B.*) *microplus* larvae were only repelled by 10% lavender oil preparations (Fig. 3d). Whether specific compounds within these essential oils would be more potent remains to be determined.

This study highlights the potential of three commercially available plant-derived compounds for the control of the cattle fever tick, *R.* (*B.*) *microplus*. Our data demonstrate that Stop the Bites is an effective acaricide against *R.* (*B.*) *microplus* larvae and adults, greatly reducing their survival at low concentrations and significantly decreasing their reproductive potential. NootkaShield and BioUD also significantly diminished the reproductive potential of *R.* (*B.*) *microplus* females at concentrations of 5%. Although on-host assays must be performed to evaluate their potential use in the management of *R.* (*B.*) *microplus* under field conditions, these results represent a promising first step. The addition of plant-derived alternatives for the control of *R.* (*B.*) *microplus* would not only provide acaricidal compounds that can be rotated within integrated tick management programs.

## Data Availability

All data and materials reported in this publication are available upon request.
